# Effects of Stimulus Timing on the Acquisition of an Olfactory Working Memory Task in Head-Fixed Mice

**DOI:** 10.1523/JNEUROSCI.1636-22.2023

**Published:** 2023-04-26

**Authors:** Josefine Reuschenbach, Janine K. Reinert, Xiaochen Fu, Izumi Fukunaga

**Affiliations:** Sensory and Behavioural Neuroscience Unit, Okinawa Institute of Science and Technology Graduate University, Tancha, Onna, Okinawa 907-0497, Japan

**Keywords:** learning, olfaction, speed-accuracy trade-off, working memory

## Abstract

Acquisition of a behavioral task is influenced by many factors. The relative timing of stimuli is such a factor and is especially relevant for tasks relying on short-term memory, like working memory paradigms, because of the constant evolution and decay of neuronal activity evoked by stimuli. Here, we assess two aspects of stimulus timing on the acquisition of an olfactory delayed nonmatch-to-sample (DNMS) task. We demonstrate that head-fixed male mice learn to perform the task more quickly when the initial training uses a shorter sample-test odor delay without detectable loss of generalizability. Unexpectedly, we observed a slower task acquisition when the odor–reward interval was shorter. The effect of early reward timing was accompanied by a shortening of reaction times and more frequent sporadic licking. Analysis of this result using a drift-diffusion model indicated that a primary consequence of early reward delivery is a lowered threshold to act, or a lower decision bound. Because an accurate performance with a lower decision bound requires greater discriminability in the sensory representations, this may underlie the slower learning rate with early reward arrival. Together, our results reflect the possible effects of stimulus timing on stimulus encoding and its consequence on the acquisition of a complex task.

**SIGNIFICANCE STATEMENT** This study describes how head-fixed mice acquire a working memory task (olfactory delayed nonmatch-to-sample task). We simplified and optimized the stimulus timing, allowing robust and efficient training of head-fixed mice. Unexpectedly, we found that early reward timing leads to slower learning. Analysis of this data using a computational model (drift-diffusion model) revealed that the reward timing affects the behavioral threshold, or how quickly animals respond to a stimulus. But, to still be accurate with early reaction times, the sensory representation needs to become even more refined. This may explain the slower learning rate with early reward timing.

## Introduction

How animals learn is a question that has long intrigued humankind (Hume, 1748; Thorndike, 1898; Pavlov, 1927; Skinner, 1950). Systematic studies of behavioral changes resulting from stimulus associations have enabled quantitative descriptions of relationships between physical stimuli and behavior, and in some cases, allowed identification of corresponding physiological mechanisms (Milner et al., 1998). Insights from such animal experiments have an impact on human interactions, too, as seen in their influence on educational psychology (Shuell, 1986; Kay and Kibble, 2016). Decades of neurophysiological studies have revealed that neuronal activity patterns evoked by sensory stimuli are highly dynamic, evolving over time in information content and eventually decaying to spontaneous patterns of activity (Fairhall et al., 2001; Friedrich and Wiechert, 2014). As a result, the stimulus timing used in behavioral training may fundamentally govern the patterns of neural activity that are reinforced. Therefore, a careful characterization of how stimulus timing affects the acquisition of sensory-guided tasks is crucial for understanding reinforcement learning.

Delayed match-to-sample (DMS)/Delayed nonmatch-to-sample (DNMS) tasks are relatively complex tasks, in that the association formed is not simply between one stimulus with a reward (Skinner, 1950; Blough, 1959). In these paradigms, animals are presented with two stimuli (sample and test stimuli), separated by an interval (delay), and the task for the animals is to report if the identities of the two stimuli match in the case of the DMS task, or are different, in the case of the DNMS task. These paradigms have become essential for studying working memory in various model organisms, ranging from pigeons and dolphins to primates and humans (Skinner, 1950; Blough, 1959; Herman and Gordon, 1974; Mishkin and Delacour, 1975; Hampson et al., 1993). They have also been used to probe the perceptual similarities of sensory stimuli (Zentall and Smith, 2016; Nakayama et al., 2022) and continue to reveal insights into brain functions, such as the roles played by the sensory and prefrontal cortices (Liu et al., 2014; Eriksson et al., 2015; Zhang et al., 2019; Wu et al., 2020) and the limbic system (Mishkin and Manning, 1978; Hampson et al., 1993; Eichenbaum et al., 2007) in retaining information over time. Previous studies succeeded in training head-fixed mice to perform olfactory DMS/DNMS tasks (Liu et al., 2014; Han et al., 2018; Zhang et al., 2019; Wu et al., 2020; Nakayama et al., 2022), although the training protocols described vary considerably.

In this study, we wished to understand how two aspects of stimulus timing affect the acquisition of an olfactory DNMS task. First, we assess the effect of the interodor interval on the rate of DNMS acquisition. This is a parameter unique to working memory paradigms like DNMS tasks. As this type of short-term memory decays over time (Baddeley, 2012), DNMS task acquisition is likely more robust with a shorter sample-test interval, although without the guarantee that the performance generalizes well to longer intervals. In addition, we assess the effect of reward timing. It is commonly accepted that a shorter delay of reinforcement is more effective for learning (Renner, 1964), with more discounting to take place for longer delays (Mazur, 1987; Myerson and Green, 1995; Calvert et al., 2010). More modern reinforcement learning models incorporate such discounting as a weakening of the eligibility trace over time (Barto et al., 1983).

We demonstrate that the DNMS task acquisition is quicker and without detectable loss of generalizability when the initial training uses a shorter interodor delay. Unexpectedly, we observed a slower task acquisition with a shorter stimulus–reward interval. In combination with a drift-diffusion model, we reveal that a primary characteristic of early reward delivery during training is a lowering of the decision bound. This lower bound requires greater discriminability in the sensory representations to achieve the same behavioral accuracy, which may underlie the slower learning rate. Together, our results reflect possible consequences of stimulus timings on the quality of stimulus encoding and the acquisition of complex behavioral tasks.

## Materials and Methods

### Animals

All animal experiments had been approved by the Okinawa Institute of Science and Technology Graduate University Graduate Animal Care and Use Committee (Protocol 2021–350). C57BL6J mice were used for the DNMS training, and a mixture of C57BL6J and wild-type mice from Ai39 breeding were used for the mixture discrimination training. The genotypes were balanced across experimental groups. C57BL6J mice were purchased from Japan CLEA and were acclimatized to the facility for at least 1 week before they were used for experiments. All mice used in this study were adult male (8–11 weeks old at the time of head plate implantation). Mice were randomly distributed across cohorts, and times of training were matched across experimental groups.

### Olfactometry

**Figure 1.**
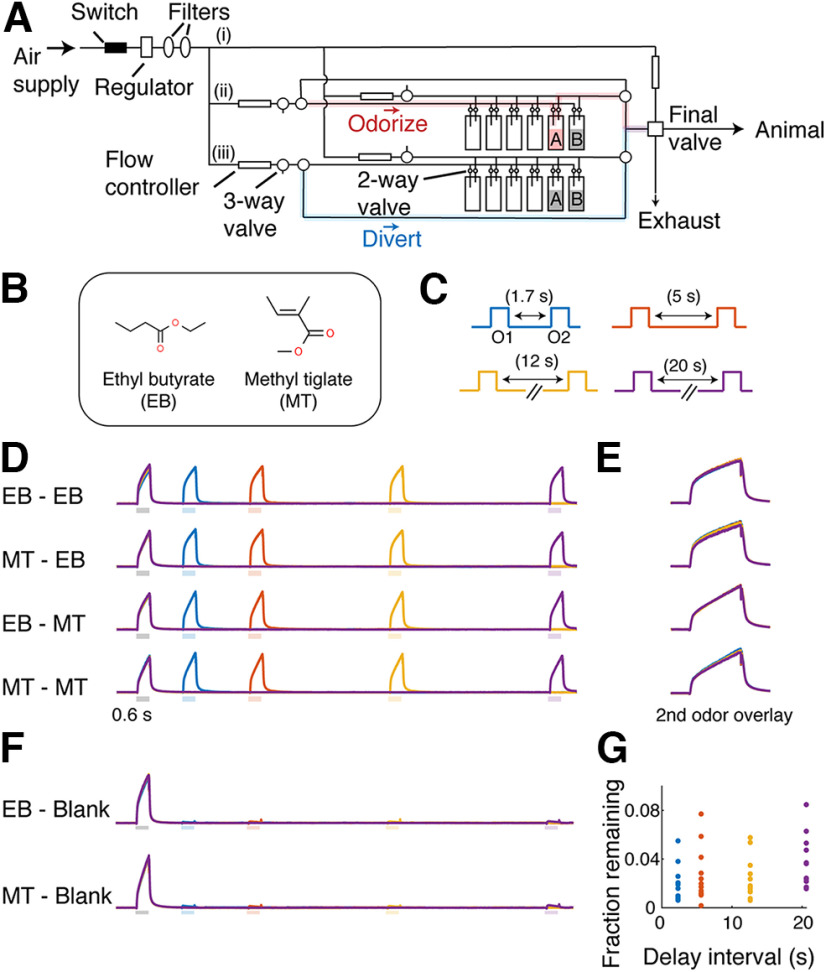
An olfactometer design for stable odor presentations. ***A***, A schematic of the design. Filtered air supply is split into three paths, (i) a stream that normally flows to the animal, (ii) a stream used for the sample odor, and (iii) a stream used for the test odor. Each odorizing stream has a pair of three-way valves. When one path is engaged for odor presentation, the air from the selected odor path passes through an odor canister before being directed toward the final valve (red highlight). Simultaneously, the other path diverts air directly toward the final valve (blue highlight), bypassing the odor canisters. ***B***, Odors used in this paradigm. ***C***, Four sample odor (O1) test odor (O2) delays used for assessing the olfactometer performance, which were randomly interleaved. ***D***, Example photoionization detector signals for the four permutations of sample and test odors (average of 12 trials), color coded by the interval used as in ***C***. ***E***, Photoionization detector signals for the test odor overlaid for all intervals tested. ***F***, Test odors were passed through blank odor canisters to test the level of cross-contamination at four delay intervals as in ***C***. ***G***, Photoionization detector signals during the test stimulus periods were expressed as a fraction of the sample odor signal level. Levels from individual trials are shown.

A custom-made flow-dilution olfactometer was used to present odors (Fig. 1). Briefly, custom LabVIEW codes were used to control (1) sourcing output digital output modules (NI-9474, National Instruments), which actuated direct-operated solenoid valves, used to control air flow and gate reward delivery (water droplets); (2) analog output modules (NI-9263, National Instruments) to generate signals for flow controllers (C1005-4S2-2L-N2, FCON) and to communicate the output trial types with acquisition boards; and (3) digital input/output modules (NI-9402, National Instruments) to control LEDs and to communicate valve opening timing.

A pair of normally closed solenoid valves was assigned per odorant to odorize the air stream. These solenoid valves were attached to a manifold so that a set of eight pairs connected to the common air stream (Fig. 1). On each trial, the sample and test odors came from two separate manifolds to avoid cross-contamination. Further, each odorant was duplicated on two manifolds to allow presentation of a particular odor from either manifold, avoiding an auditory association for the behavioral task. An air stream was odorized only when a pair of three-way valves was actuated and directed toward the animal when the solenoid valve closest to the animal (final valve) opened. The final valve was opened for 0.6 s for each odor presentation. Odors were presented at ∼1% of the saturated vapor. Total air flow, which is a sum of odorized air and the dilution air, was ∼2 L/min, which was matched by the air that normally flows toward the animal. The intertrial interval was ∼40 s to purge the airways to minimize cross-contamination. Ethyl butyrate (EB; catalog #W242705), ethyl valerate (catalog #290866), and butyl acetate (catalog #287725) were purchased from Sigma-Aldrich, and methyl tiglate (MT; catalog #T0248), methyl valerate (catalog #S0015), methyl salicylate (catalog #V0005), and eugenol (catalog #A0232) were purchased from Tokyo Chemical Industry. Stock odorants were stored at room temperature in a cabinet filled with N_2_ and away from light.

### Surgery

All recovery surgeries were conducted in an aseptic condition. For head plate implantation, 8- to 11-week-old male C57BL6/J mice were deeply anesthetized with isoflurane. The body temperature was kept at 36.5°C using a heating blanket with a DC controller (FHC). To attach a custom head plate ∼1 cm in width weighing ∼1 g, the skin over the parietal bones was excised, and the soft tissue underneath was cleaned, exposing the skull. The exposed skull was gently scarred with a dental drill, cleaned, dried, and coated with cyanoacrylate (Histoacryl, B. Braun) before placing the head plate, which was fixed in place with dental cement (Kulzer). Mice were recovered in a warm chamber, returned to their cages, and given carprofen subcutaneously (5 mg/kg) for 3 consecutive days.

### Habituation and behavioral measurements

Water restriction began 2–3 weeks after surgery. Mice went through habituation to head fixation, one session per day for ∼30 min. No odors were presented. This lasted at least 3 d or until the mice learned to lick vigorously for a total water reward of at least 1 ml. The respiration pattern was measured by sensing the airflow just outside the right nostril by placing a flow sensor (AWM3100V, Honeywell). Lick responses were measured using an infrared beam sensor (PM-F25, Panasonic) that was a part of the water port. Nasal flow, an analog signal indicating the odors used, lick signal, a copy of the final valve, and water valve timing were acquired using a multifunction input/output device (USB-6363, National Instruments).

### Olfactory DNMS training

After habituation, the head-fixed mice were trained to associate a water reward with a nonmatching combination of odors (ethyl butyrate and methyl tiglate). Each trial started with brief flashes of a blue LED (8 rectangular pulses at 8 Hz, 50% duty cycle). The reward comprised two droplets of water (10 µl each) that arrived on all rewarded trials after the onset of the final valve opening. Anticipatory licks were defined as licks before the water delivery in response to the second odor presentation. Correct reactions were defined as the generation of anticipatory licks in response to nonmatching odor pairs and a lack of licking in response to matching odor pairs. Once the overall accuracy for rewarded and unrewarded trials was above 80% in at least one behavioral session, the delay interval was increased. A typical training session comprised ∼100 trials, lasting ∼1 h. Rewarded and unrewarded trials occurred at equal probability, where the specific permutation of the odors used (e.g., EB-MT or MT-EB for rewarded trials) was randomly assigned. To deliver the reward with a timing that depended on the timing of anticipatory licks of the mouse, lick signals were analyzed online for a brief period following the test odor presentation (0.6–3 s after the test odor onset). The reward was delivered on rewarded trials when the number of anticipatory licks exceeded a threshold. The threshold for triggering an output here was equivalent to three licks. The latest reward delivery was at 3 s. Interval between the test odor onset and the start of the next LED signal was ∼18 s. All data were acquired at 1 kHz.

### Olfactory discrimination training

The olfactory discrimination training was adapted from Koldaeva (2019). After habituation, the head-fixed mice were trained to discriminate two binary odor mixtures of ethyl butyrate and eugenol using the Go/No-Go paradigm. The training includes two stages, a discrimination between 80/20 and 20/80 mixtures and a more difficult discrimination of 60/40 and 40/60 mixtures. Similar to our DNMS training, the mouse proceeded to the second stage when the overall accuracy in the first task reached 80%. Reward was two drops of water (10 µl each), dispensed unconditionally (every rewarded trial) at 1.2 s after the odor onset for one group (early reward group, 6 mice) and 3.2 s for the second group (late reward group, 6 mice).

### Odor similarity measures

The similarity between a pair of monomolecular odors was determined by calculating the correlation coefficient of population activity from extracellular recording from the olfactory bulb of anesthetized mice (ketamine/xylazine, 100 mg/kg and 20 mg/kg, respectively). To make the craniotomy, male adult Bl6 mice (8–12 weeks old) were anesthetized with isoflurane and kept warm with a temperature-controlled blanket. The skin overlying the frontal and nasal plates were excised, the underlying periosteum was scraped, and a head plate secured with cyanoacrylate gel (Loctite) and dental acrylic. One small craniotomy for the earth connector was made in the caudal part of the right frontal plate. A 0.2-mm-diameter craniotomy for electrophysiology was made over the left olfactory bulb. The approximate stereotaxic coordinate for the center of this craniotomy was 4.5/1 (AP/ML) relative to Bregma. Once the craniotomy was made, isoflurane was removed, and ketamine/xylazine was injected intraperitoneally immediately. Extracellular recording was obtained with a 16-channel silicon probe (A1x16-Poly 2-5 mm-50 s-177, catalog #CM16LP) acquired at 25 kHz with a low-noise amplifier (RHD2132) and RHD controller C3004 (Intan Technologies). Probes were advanced vertically, and units were encountered at the tip depth of ∼600 µm relative to the brain surface (*n* = 7 locations, 3 mice). Data were high-pass filtered and units sorted off-line using Kilosort 2.0 software (Pachitariu et al., 2016; Stringer et al., 2019). Parameters are adapted from the recommended parameters from Kilosort (ops.fs = 20,000; ops.fshigh = 300; ops.minfr_goodchannels = 0; ops.Th = [10 4]; ops.lam = 10; ops.AUCsplit = 0.9; ops.minFR = 1/50; ops.momentum = [20 400]; ops.sigmaMask = 30; ops.ThPre = 8;). The channel map was recreated from the layout of the recording electrode (A1x16-Poly2s, NeuroNexus). Then the automated outputs from Kilosort were manually curated with Phy, an open-source graphical user interface (Rossant et al., 2016). Only those clusters with a clear refractory period, contamination percentage less than 20%, and with waveforms distinguishable from the background were labeled as good single units and used for further analysis.

### Experimental design and statistical analyses

A full set of descriptions on the statistical tests used, along with the test statistics and sample sizes, is available in Table 1.

**Table 1.** Statistical tests and their details

Location in article	Test used	Sample size	Test statistic	Exact *p* value
Figure 2 *F*	Pearson correlation coefficient	112 Blocks from 6 mice	*r* = −0.785	1.1731 × 10^−24^
Figure 3*C* (left)	One-way ANOVA with *post hoc* multiple comparisons (Tukey–Kramer)	Two groups (6 mice and 5 mice)	*F* = 4.26	0.0378
Figure 3*C* (right)	One-way ANOVA with *post hoc* multiple comparisons (Tukey–Kramer)	Two groups (6 mice and 5 mice)	*F* = 5.82	0.0157
Figure 3 *D*	Pearson correlation coefficient	61 Blocks from 5 mice	*r* = −0.549	4.727 × 10^−6^
Fig. 4*F* (left)	two-sample *t* test	Two groups (5 mice and 6 mice)	*t* = 2.3014	0.0469
Fig. 4*F* (middle)	two-sample *t* test	Two groups (5 mice and 6 mice)	*t* = 2.0727	0.0681
Fig. 4*F* (right)	two-sample *t* test	Two groups (5 mice and 6 mice)	*t* = −0.1736	0.8665
Figure 4 *I*	Pearson correlation coefficient	55 Blocks from 6 mice	*r* = −0.483	1.833 × 10^−4^
Figure 5 *A*	One-way ANOVA with *post hoc* multiple comparisons (Tukey–Kramer)	Three groups (6 mice, 5 mice, 6 mice)	*F* = 4.25	0.036
Figure 5 *B*	One-way ANOVA with *post hoc* multiple comparisons (Tukey–Kramer)	Three groups (6 mice, 5 mice, 6 mice)	*F* = 4.12	0.0393
Figure 5*C* (left)	One-way ANOVA with *post hoc* multiple comparisons (Tukey–Kramer)	Two groups (5 mice and 6 mice)	*F* = 5.38	0.0455
Figure 5*C* (right)	One-way ANOVA with *post hoc* multiple comparisons (Tukey–Kramer)	Three groups (6 mice, 5 mice, 6 mice)	*F* = 3.75	0.0498
Figure 6 *F*	*t* Test with Bonferroni multiple-comparison correction	Seven mice	*t* Statistics (1.7 s, 5 s, 8 s, 12 s, 20 s) = 7.3560, 8.6679, 6.5347, 5.2867, 3.4159)	0.0007, 0.0003, 0.0013, 0.0032, 0.0189
Fig. 9*E* (top)	One-way ANOVA with *post hoc* multiple comparisons (Tukey-Kramer)	Three groups (6 mice, 5 mice, 6 mice)	*F* = 6.0588	0.0127
Fig. 9*E* (bottom)	One-way ANOVA with *post hoc* multiple comparisons (Tukey–Kramer)	Three groups (6 mice, 5 mice, 6 mice)	*F* = 5.5841	0.0165
Fig. 10 *F*	Two-sample *t* test	Six mice in each cohort	*t* = −2.9758	0.0139

### Data analysis

#### Analysis of the behavioral data

The behavioral data were analyzed using built-in event detection functions in the Spike2 package to obtain valve opening times and were further analyzed in MATLAB using custom codes. The number of anticipatory licks after the test odor offset was counted for each trial to calculate the accuracy. When the reward timing was variable, the temporal window analyzed was until the onset of the water valve opening. To analyze comparable temporal windows across trial types, for unrewarded trials, an average water valve timing from the rewarded trials was used. For fixed reward timing, a 2.6 s window from the test odor offset was used. Using a shorter window to mimic the condition for the variable reward timing did not affect the result. To calculate the learning curve, the accuracy was expressed as the area under the receiver operating characteristic (auROC) in a given block of 50 trials, using the MATLAB function perfcurve.

### Dependence of DNMS performance on delay interval

To analyze how the accuracy (auROC) of DNMS task performance depended on the interodor interval (10) in a session where the delay interval varied from trial to trial, mean auROC values across animals were fitted using a single-term exponential using the MATLAB fit function with the function set to exp1, which estimates the parameters α and λ for the following equation: $accuracy(x)=\alpha e^{-\lambda x}.$

#### Trials to criterion

For each animal, block-by-block auROC values were fitted with a logistic function, $p(n)=\frac{1}{1+e^{-(\beta_{0}+\beta_{1}n)}},$ where $\beta_{0}$ is known as the intercept, and $\beta_{1}$/4 was used to estimate the slope at the steepest point (Gelman and Hill, 2006). Using this fit, the number of trials required to reach auROC value of 0.8 was interpolated for each animal.

#### Reaction time

Reaction time is equivalent to the response time, which was calculated by measuring the average timing of the first three licks after the test odor on rewarded trials. It is expressed relative to the onset of the test odor.

#### Stray licks

All licks between the onsets of the sample and test odors were counted and divided by this interval.

#### Probability of false alarms

Lick occurrences following the test odor presentation (0–3 s relative to the onset) on unrewarded trials were detected and used to construct peristimulus time histograms with a bin size of 0.02 s, which was normalized by the number of trials.

### Drift-diffusion model

The reaction times were modeled using a simple drift-diffusion model based on Ratcliff and McKoon (2008) but adapted for the Go/No-Go paradigm as described in Ratcliff et al. (2018). The model is described by three parameters, the Go decision bound and two instantaneous drift rates, µ_s−_, for match (S−) and µ_s+_ for nonmatch (S+) trials. At time *t* = 0, the accumulated evidence was set to 0.5 for all data. Time steps were discretized in 20 ms (Δt). For each new time step, new momentary evidence was drawn from a stationary distribution, x(Δt) = N(µ_i_,1), a normally distributed random generator with a mean of µ_i_, where i = [S+, S−], and a noise parameter of 1 SD. This was accumulated over time to yield the following sensory evidence: $s(n\Delta t)=\overset{n}{\underset{j=1}{\sum }}x(\Delta t).$ The reaction time was defined as the time at which this sensory evidence crossed the decision bound. The parameters were obtained for each animal by fitting the data from each behavioral session by minimizing the chi-square values (Ratcliff et al., 2018).

## Results

In the DNMS task, two stimuli (sample and test) are presented with a delay in between, and the animals are required to report when the identities of the two stimuli do not match. To implement this task using olfactory stimuli, an olfactometer should be capable of presenting stable olfactory stimuli with minimal cross-contamination, regardless of the sample-test delay durations used. To achieve this, we designed a novel olfactometer (Fig. 1; see above, Materials and Methods). Stable and clean presentations of the two odors are achieved by separating the air streams for the sample and test odors for most parts of the odor pulse preparation while allowing continuous air passages to avoid a pressure buildup.

**Figure 2.**
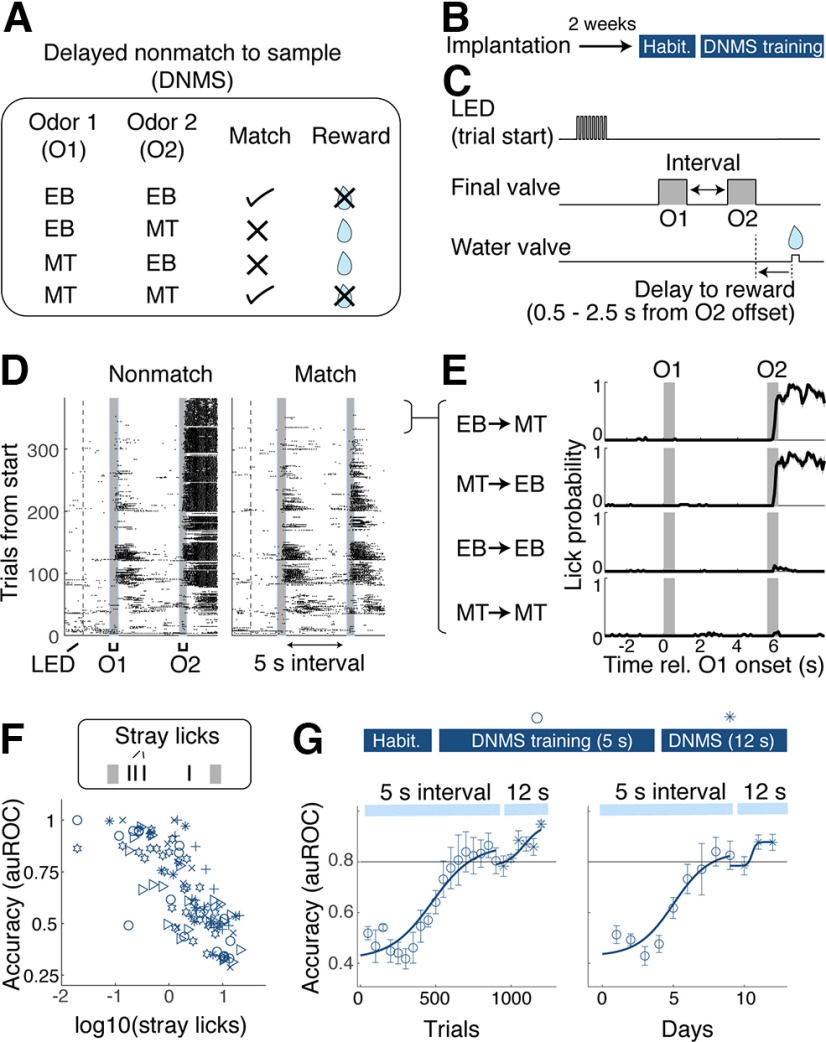
Go/No-Go olfactory delayed nonmatch-to-sample task with a 5 s delay. ***A***, Illustration of the olfactory DNMS. On a given trial, two odors (Odor 1 and Odor 2) are presented. Rewarded trials are trials where two odors are not the same (nonmatch). Reward is 20 µl of water. ***B***, Timeline of training. Behavioral training started 2 weeks after surgery for head plate implantation. ***C***, Schematic showing the trial structure. Flashes of an LED indicate the trial start. Sample and test odors (O1 and O2, respectively) are presented with an interval (5 s for the initial training, and 12 s afterward). Water reward was delivered on all rewarded trials. Reward was delivered earlier when mice generated anticipatory licks earlier (range of possible reward times, 0.5–2.5 s from O2 offset). ***D***, Lick raster for an example session, separated by nonmatch trials (left) versus match trials (right). ***E***, Example peristimulus time histogram of licks separated by four trial types from a proficient mouse. ***F***, Relationship between stray licks (licks during the sample-test odor interval) and accuracy of DNMS performance. Symbols indicate individual mice. ***G***, Learning curves for 5 s delay (hollow symbols) and 12 s delay (stars) shown with respect to the trials from the start of training (block size, 50 trials; left) and with respect to days (right); *n* = 6 mice. Mean and SEM are indicated.

Using the new olfactometer, we tested whether a simplified olfactory DNMS training leads to efficient and robust learning without the need for autoassociation phases (Liu et al., 2014; Han et al., 2018; Nakayama et al., 2022), punishments (Wu et al., 2020), and multiple training sessions per day (Han et al., 2018) used in some implementations. Mice were subjected to olfactory DNMS training immediately after habituation (Fig. 2*A*,*B*). The two odors used in this paradigm were EB and MT. On each trial, the identities of sample and test odors were randomly chosen and presented to the animal with a 5 s delay between odors (Fig. 2*C*). When the sample and test odors did not match (nonmatch trial), a reward (2 droplets of 10 µl water) was delivered unconditionally, with a default delivery at 2.5 s after the test odor offset. However, the reward was delivered earlier if the mice produced early and vigorous anticipatory licks, with the earliest delivery at 0.5 s after the test odor offset. On average, the reward was dispensed when three anticipatory licks were detected (see above, Materials and Methods).

In the initial phase of the training, the mice generated many sporadic licks, regardless of the sample and test odor combinations and also during the sample-test odor delay (Fig. 2*D*). However, with training, mice learned to produce anticipatory licks more selectively in response to nonmatching sample and test odor combinations (Fig. 2*E–G*). They reached a criterion level of performance (auROC = 0.8) on average within 705 ± 83.6 trials, or 7.2 ± 0.7 d (mean ± SEM; *n* = 6 mice). Notably, other undesirable licks, such as licks during the sample-test delay period, decreased in the absence of punishments (average number of licks during 5 s delay = 7.13 ± 1.60 for the first 2 d vs 0.85 ± 0.39 for the last 2 d of training; *p* = 0.016, paired *t* test; *n* = 6 mice). In general, the reduction in these sporadic licks and the behavioral accuracy of the task developed together (Fig. 2*D–F*; Pearson correlation coefficient = −0.785, *p* = 1.17 × 10^−24^; *n* = 112 blocks of 50 trials analyzed from 6 mice), suggesting that the loss of sporadic licks may occur as a natural consequence of learning the concept of the task. Overall, this learning rate is comparable to previous reports of olfactory DNMS paradigms (Han et al., 2018), especially when the shaping phase of those training paradigms is considered. Further, when the sample-test delay period was increased to 12 s, mice performed significantly above chance from the first session (Fig. 2*G*; mean accuracy in auROC = 0.848 ± 0.026; *p* = 3.81 × 10^−5^; *n* = 6 mice; Student's *t* test for mean auROC = 0.5), demonstrating that the acquired behavior is generalizable across delay intervals. Overall, the results indicate that a simplified training leads to a robust acquisition of DNMS performance.

**Figure 3.**
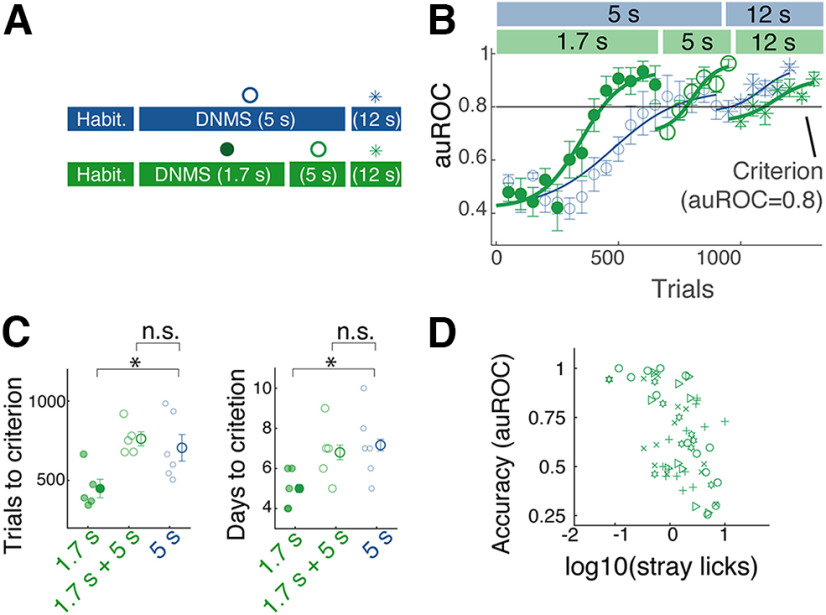
DNMS with a shorter interval is easier to learn. ***A***, Two training designs are compared. One cohort of mice that started the training with sample-test odor interval of 1.7 s (green) was compared with those that started with 5 s interval (blue; same data as Fig. 2). ***B***, Learning curves for the two cohorts, showing behavioral accuracy in auROC for for 1.7 s delay (filled circles), 5 s delay (hollow circles), and 12 s delay (stars). Color scheme as in ***A***. Block size, 50 trials; *n* = 6 and 5 mice for initial training with 5 s and 1.7 s, respectively. ***C***, Summary comparison of acquisition speeds in terms of number of trials (left) and number of days (right) taken to reach the criterion (auROC = 0.8); *p = 0.043 (left) and 0.036 (right), one-way ANOVA with *post hoc* Tukey–Kramer comparisons; n.s., *p* = 0.82 (left) and 0.80 (right). Mean and SEM are indicated. ***D***, Relationship between stray licks (licks during the sample-test odor interval) and accuracy of DNMS performance. Pearson's *r* = −0.549, *p* = 4.73 × 10^−6^. Symbols correspond to individual mice. n.s. = not significant at the 0.05 level.

We next assessed whether the sample-test odor delay duration affects the initial acquisition of the task (Fig. 3). The second cohort of mice was trained with the DNMS task with a shorter sample-test odor delay of 1.7 s. All other aspects were held the same as before (Fig. 3*A*). The mice reached the criterion level of performance, on average, in 449.0 ± 58.3 trials (mean 5 ± 1 d), which is significantly faster than with the 5 s delay (Fig. 3*B*,*C*; *p* = 0.039, *t* test; *n* = 6 and 5 mice for 5 s delay and 1.7 s delay, respectively). Again, when the sample-test odor interval was increased to 5 s and 12 s, the performance accuracy was significantly above chance from the beginning (average accuracy on the first day = 0.87 ± 0.01 auROC for 5 s and 0.81 ± 0.02 for 12 s; *p* = 1.53 × 10^−5^ and 1.57 × 10^−4^; Student's *t* test for auROC = 0.5; *n* = 5 mice). The two cohorts of mice reached the criterion levels of performance at 5 s and 12 s delays subsequently in comparable numbers of trials (Fig. 3*C*; data not shown for 12 s). As before, the initial acquisition of the task was accompanied by a reduction in the sporadic licking during the interodor delay period (Fig. 3*D*; Pearson correlation coefficient = −0.549, *p* = 4.73 × 10–6; *n* = 61 blocks from 5 mice). Together, these results demonstrate that acquisition of the DNMS task is more efficient with a shorter sample-test interval but with no advantage when reaching a proficient performance on a longer sample-test delay is required.

**Figure 4.**
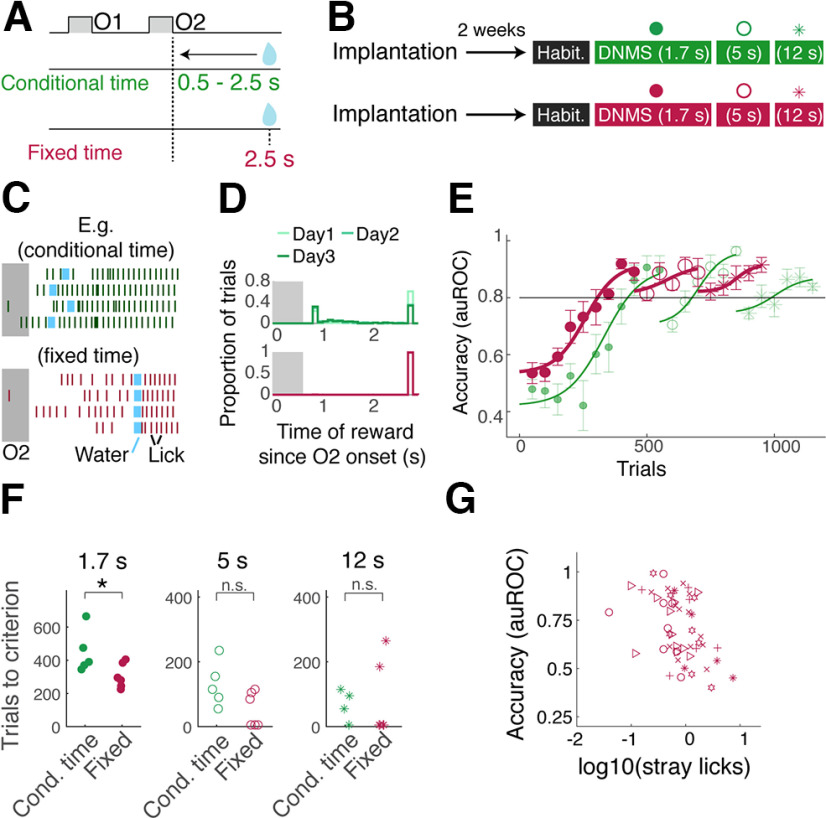
Effect of reward timing on DNMS acquisition. ***A***, With conditional reward timing (green), the water reward was delivered earlier if mice licked earlier (possible reward onset ranged from 0.5 to 2.5 s after the odor 2 offset). With fixed reward delivery time (burgundy), water reward was delivered at 2.5 s after the odor 2 offset. ***B***, Timeline of training for the two cohorts, with sample-test odor interval indicated inside the parentheses. ***C***, Examples of lick timing (vertical lines) relative to odor 2 (duration 0.6 s) and water delivery (light blue). For the conditional reward time group (left), the timing of reward onset depended on lick timing. For the fixed reward onset (right), water timing was fixed regardless of licking behavior. ***D***, Histograms of water onset times for the two groups, for days 1–3 of the training. ***E***, DNMS learning curves for the two cohorts for 1.7 s (filled circles), 5 s (hollow circles), and 12 s (stars) intervals. Horizontal line indicates the criterion level (auROC = 0.8). ***F***, Summary of learning speeds, measured by number of trials taken to reach criterion performance at sample-test odor interval of 1.7 s (left), 5 s (middle), and 12 s (right) for the two cohorts; *p* = 0.047, 0.068, 0.87 (two-sample *t* tests), respectively; *n* = 5 mice for conditional reward timing and 6 mice for fixed reward timing. ***G***, Relationship between stray licks (licks during the sample-test odor interval) and accuracy of DNMS performance. Pearson's *r* = −0.483, *p* = 1.83 × 10^−4^. Symbols correspond to individual mice. Cond., Conditional. n.s. = not significant at the 0.05 level.

In the above training protocols, the reward was delivered earlier when the mice licked earlier to motivate them to perform the task. To determine what influence this reward timing has on the DNMS task acquisition, the third cohort of mice was trained with a fixed reward time while keeping other factors constant, with the initial sample-test delay set to 1.7 s (Fig. 4*A*,*B*). The reward (2 × 10 µl water) was delivered 2.5 s after the offset of the test odor regardless of when animals generated anticipatory licks (Fig. 4*C*,*D*). On average, the reward was delivered 1.35 ± 0.13 s later for this cohort than for the previous group (*p* = 6.99 × 10^−7^, one-way ANOVA; *n* = 5 and 6 mice for the conditional and fixed reward time groups, respectively). Surprisingly, the learning curves of the two cohorts revealed that the mice that received the reward at a fixed time acquired the task faster than the cohort with conditional timing (Fig. 4*E*,*F*; trials to criterion 305.8 ± 30.1 trials, compared with 449.0 ± 58.3 trials for the group with conditional reward timing). However, their ability to perform at longer sample-test odor intervals was comparable (average accuracy for the initial sessions = 0.87 ± 0.02 and 0.87 ± 0.03 for 5 s and 12 s intervals, respectively; *p* = 0.22 and 0.19, two-way ANOVA for the effect of cohort and interval durations, respectively). Again, the amount of sporadic licking negatively correlated with the behavioral accuracy (Fig. 4*I*). Overall, the result indicates that the reward timing affects the task acquisition, revealing an unexpected and detrimental effect with the early reward delivery, without affecting the generalizability of performance across intervals.

**Figure 5.**
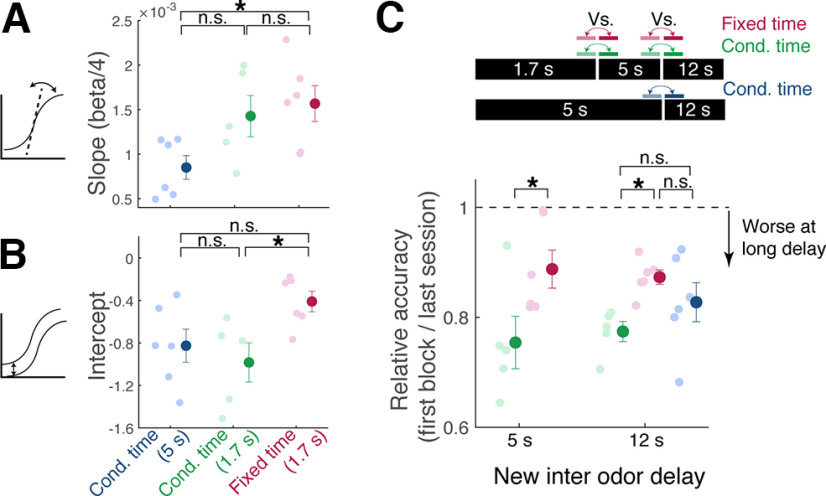
Olfactory stimulus interval affects the slope, whereas reward timing affects the intercept of learning curves. ***A***, Slope of the logistic curve, derived from the fitted parameter, beta, for the three protocols used. Beta/4 describes the slope at the steepest point; *p* = 0.036, one-way ANOVA, followed by Tukey–Kramer *post hoc* comparisons (*p* = 0.1202, 0.0377, and 0.8662 for 1 vs 2, 2 vs 3, and 1 vs 3 comparisons). ***B***, Intercept values for the fitted logistic curves; *p* = 0.0393, one-way ANOVA, followed by Tukey–Kramer *post hoc* comparisons (*p* = 0.7387, 0.1307, and 0.0405 for 1 vs 2, 2 vs 3, and 1 vs 3 comparisons). ***C***, Initial accuracy when switching to longer sample-test interval, normalized by the last session performance; *p* = 0.0455 for equal performance at 5 s interval, one-way ANOVA; *p* = 0.0498 for equal performance at 12 s, one-way ANOVA with *post hoc* Tukey–Kramer comparisons; *n* = 6 mice, 5 mice, and 6 mice for cohorts 1, 2, and 3, respectively. Cond., Conditional. n.s. = not significant at the 0.05 level.

We wished to understand in detail how the three training protocols affected the task acquisition. To do so, we fitted the behavioral accuracy over time with a logistic regression (Fig. 5*A*,*B*). This regression analysis revealed that the DNMS task acquisition with a 5 s interodor delay results in a shallower slope in the learning curve (Fig. 5*A*), indicating that a longer interval makes the task harder. On the other hand, the effect of reward timing is characterized by the lower intercept. The cohort with a late reward arrival learned to produce anticipatory licks more selectively in rewarded trials early (Fig. 5*B*). We wished to assess whether the three protocols resulted in different abilities to generalize the behavioral performance to longer intervals, which is harder as working memory tends to degrade over time (Blough, 1959; Baddeley, 2012; Liu et al., 2014). When switching to a longer interodor delay, all cohorts of mice showed an initial decline in the behavioral accuracy. Interestingly, the reward timing affected how well the mice performed at longer interodor delay intervals. The cohort of mice that was rewarded with the fixed and late timing performed better when switched to longer intervals (Fig. 5*C*).

**Figure 6.**
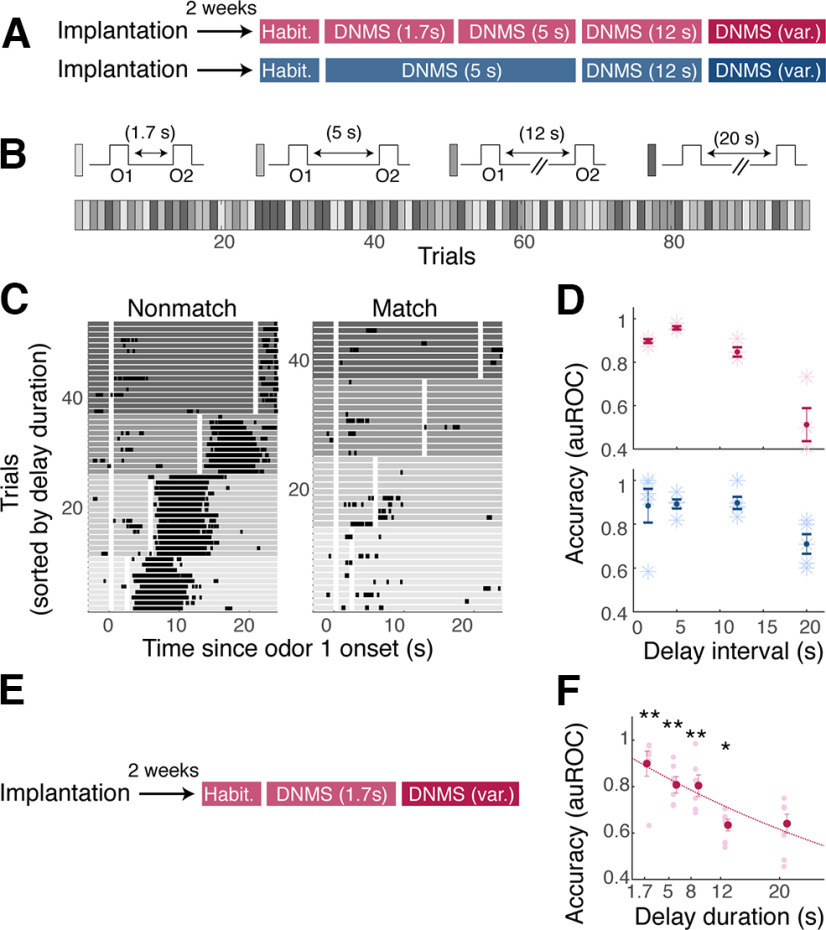
Interval-dependent performance and generalization. ***A***, Once mice were trained to perform DNMS with a 12 s interval, they went through one session where the sample-test odor interval was randomly selected from 1.7 s, 5 s, 12 s, or 20 s on a given trial [DNMS (var.)]. ***B***, Example trial order, where gray intensity indicates the sample-test odor interval duration. ***C***, Lick raster, sorted by nonmatch (left) and match (right) trials and by the duration of sample-test odor intervals, with grayscale indicating the interval duration. White areas indicate the time of odor presentations. ***D***, Accuracy for each interval used, for two cohorts, with colors corresponding to the cohorts described in ***A***. Mean and SEM are indicated; *n* = 4 mice for each cohort. ***E***, After recovery from surgery, naive mice went through habituation and training for olfactory DNMS with 1.7 s interodor interval and reward delivery fixed at 2.6 s after odor offset. Once trained, the mice performed 1–2 sessions of DNMS with variable interodor delays. ***F***, Generalization to longer intervals. Accuracy of performance for the interodor intervals is indicated, expressed auROC; ** and * indicate statistically significant deviation using *t* test from auROC = 0.5 at the significance level of 0.01 and 0.05 with Bonferroni correction, respectively; *p* = 0.0007, 0.0003, 0.0013, 0.0032, and 0.0189 for 1.7 s, 5 s, 8 s, 10 s, and 20 s delay intervals, respectively; *n* = 7 mice.

Despite the initial difference in generalization above, all cohorts learned to report when odors are not matching at the 12 s delay. We then asked whether the ability of trained mice to generalize the task performance over different sample-test odor intervals depends on how they were trained (Fig. 6*A*). For each trial, the sample-test odor delay duration was randomly chosen from four possible intervals (1.7 s, 5 s, 12 s, and 20 s; Fig. 6*B*). Some interodor delay intervals used were encountered by the animals for the first time in this session. An analysis of the behavioral performance indicated that the performance was indeed poorer at longer intervals (Fig. 6*C*,*D*). However, no statistically significant difference was present across two cohorts that underwent different training paradigms (*p* = 2.55 × 10^−6^ and 0.24, two-way ANOVA for the effects of interval and training history, respectively; *n* = 4 mice for both cohorts). Of the three protocols tested, the initial DNMS task acquisition was fastest with a short interodor delay coupled with a fixed and delayed reward arrival. To assess whether mice could generalize to different delay periods immediately after initial acquisition of the task, we subjected mice trained on the 1.7 s interval to one session of olfactory DNMS with variable interodor delay (Fig. 6*E*). The mice performed significantly above chance for longer interodor delay intervals, up to 12 s delay (Fig. 6*F*), indicating they can immediately adapt to varying interodor delays.

**Figure 7.**
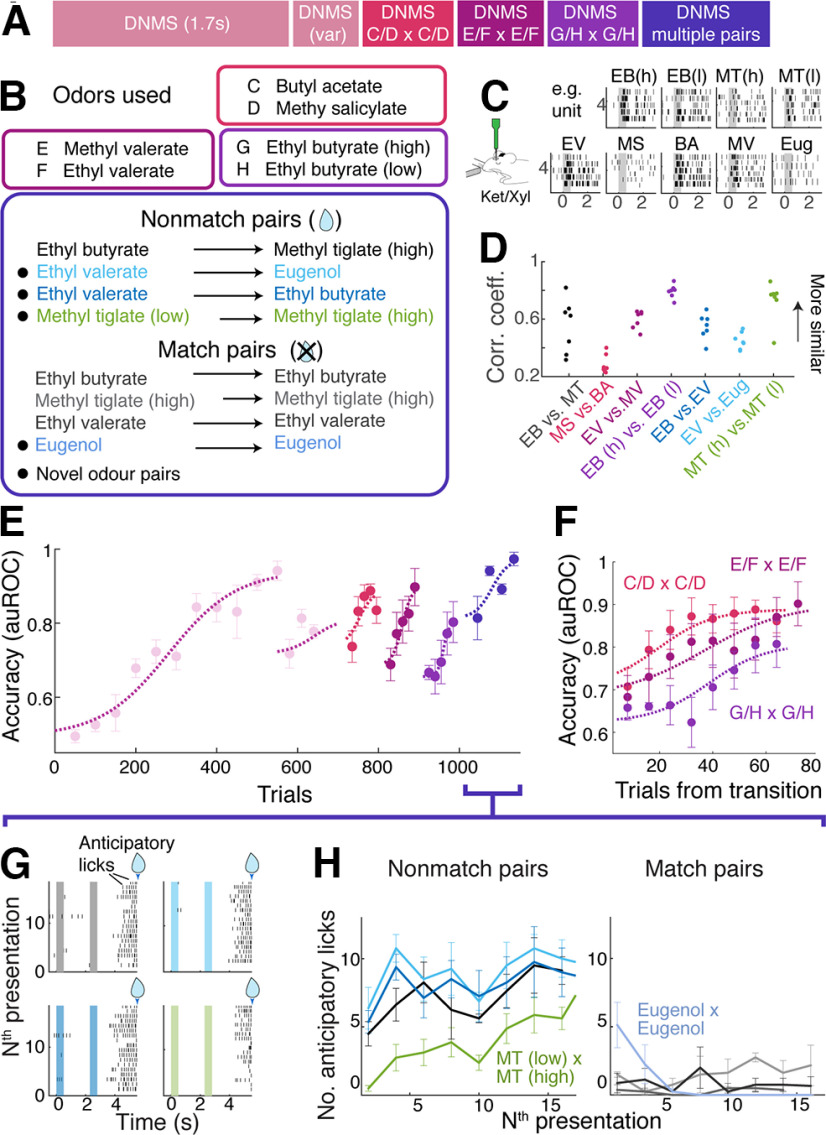
Stimulus similarity and the generalizability. ***A*, *B***, Generalization to new odor comparisons. ***A***, Timeline of behavioral training. ***B***, Odors used for the DNMS tasks, as listed in ***A***. Mice underwent DNMS training with dissimilar odor pair (butyl acetate vs methyl salicylate; C/D vs C/D), similar odor pair (ethyl valerate vs methyl valerate; E/F vs E/F), and ethyl butyrate at two concentrations (G/H vs G/H). ***C***, ***D***, Assessing the similarity of olfactory bulb activity patterns evoked in anesthetized mice by odors used in the DNMS task. ***C***, Raster plots for an example unit for all odors used. ***D***, Correlation coefficients for pairs of odors used in the DNMS tasks. Each data point corresponds to the similarity of population activity for each recording site; *n* = 7 locations, 3 mice. ***E***, Learning curves for the new odor pairs. Each data point represents the accuracy for a block of 30 trials; *n* = 4 mice. ***F***, Same data as in ***E*** but aligned for the start of training for the three new odor pairs for easy comparison. ***G,*** Lick raster plots from an example mouse for the four new nonmatch pairs introduced in the multiodor DNMS stage. Color coding follows the scheme in ***B***. ***H***, Evolution of anticipatory licks over time for new nonmatch odors (left) and match pairs (right); *n* = 4 mice. Mean and SEM are indicated.

We also used the trained mice from the above experiment to assess whether the similarity of olfactory stimuli affects the ability to generalize the task to differing stimulus difficulties. To do so, we subjected the mice initially trained to compare ethyl butyrate and methyl tiglate to new odor pairs (Fig. 7*A*). The new pairs differed in physicochemical properties (Fig. 7*B–D*). The mice were first subjected to an easily distinguishable pair (butyl acetate vs methyl salicylate), thereafter, a pair of odors that only differed by one carbon chain length (ethyl valerate vs methyl valerate), and third, a pair where the odor identity was the same but the two stimuli differed in concentrations (ethyl butyrate at high vs low concentrations; 1% vs 1.5% saturated vapor). The mice showed efficient transfer to the new odor pairs, reaching the criterion performance more quickly than it took for the initial task acquisition (Fig. 7*E,F*). There was a tendency for the mice to perform more poorly for harder pairs, but this was not statistically significant. It is possible that a sequential acquisition of odor pairs causes generalizability to improve over time. To compare the effect of stimulus similarity on the task generalizability without this confound, we subjected the same mice to multiple novel pairs within two sessions (Fig. 7*B*, bottom). This revealed that the mice learned to compare new but distinct monomolecular pairs instantaneously (Fig. 7*E*). It took the mice longer to correctly lick when they were presented with the same odor that differed in concentrations (Fig. 7*G*,*H*). Altogether, these results demonstrate that stimulus similarity affects the generalizability and speed of improvement.

**Figure 8.**
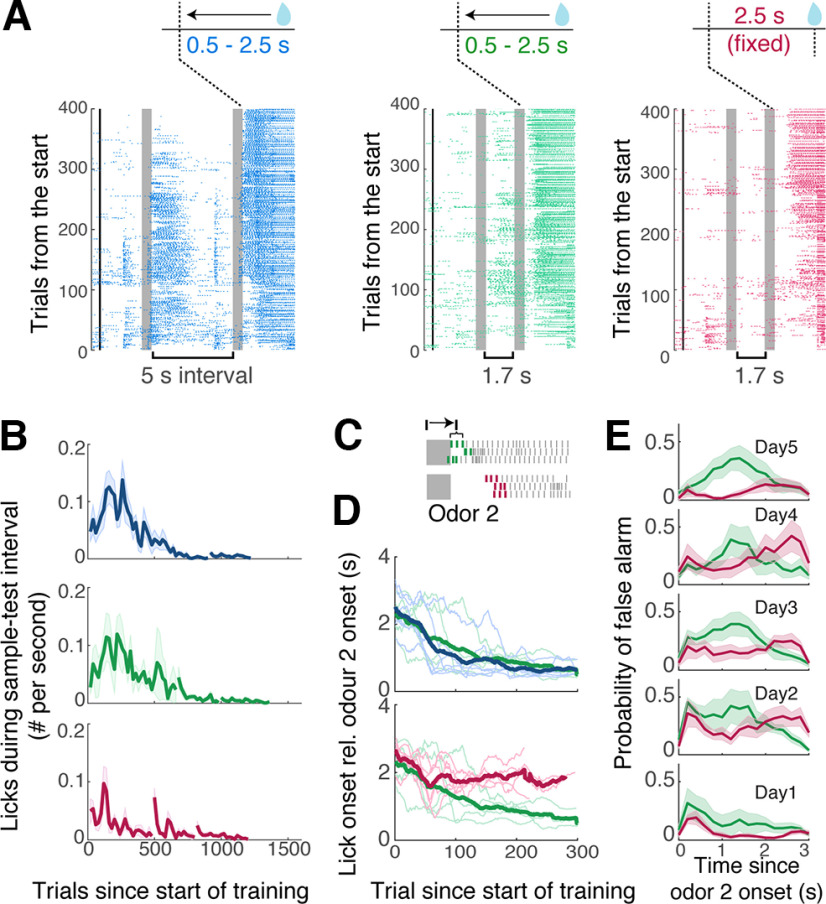
Behavioral output patterns depend on reward timing. ***A***, Example lick raster plots from mice that went through different training paradigms. Left, a mouse that started with 5 s sample-test odor interval and conditional reward timing; middle, starting with a 1.7 s interval and conditional reward timing; right, starting with a 1.7 s interval and fixed reward timing. ***B***, Evolution of stray licking during sample-test odor interval for the three cohorts (*n* = 6, 5, and 6 mice, respectively). Thick lines indicate mean; shadings indicate mean ± SEM. ***C***, Illustration of reaction times; timings of first three licks after odor 2 onset are averaged and expressed relative to the onset of odor 2. ***D***, Evolution of reaction time with training for the three cohorts indicated with the same color scheme as in ***A***. Thin lines represent individual mice, thick lines represent average. ***E***, Peristimulus time histograms for licks generated on match (unrewarded) trials were compared between cohorts that started with the 1.7 s delay but differed in the reward timing (*n* = 5 and 6 mice, respectively). Thick lines indicate mean; shading indicates mean ± SEM.

Why does the late reward arrival result in faster acquisition of the DNMS task without affecting the slope of the learning curve? To obtain clues, we first analyzed whether there was any difference in the stray lick patterns across the three cohorts during learning. We measured the stray licks generated during the sample-test odor delay (Fig. 8*A*). Among the two cohorts of mice with conditional reward timing, there tended to be more sporadic licks during the sample-test odor interval during acquisition (Fig. 8*B*; average number of stray licks per second in the first 400 trials = 1.17 ± 0.29, 1.58 ± 0.21, and 0.61 ± 0.14 for cohorts 1, 2, and 3, respectively; *p* = 0.018; one-way ANOVA; *n* = 6, 5, and 6 mice, respectively). Second, we noticed that the reaction times, that is, how quickly the mice generated anticipatory licks in response to the test odor presentation, differed between cohorts with reward timing differences. The two cohorts that could receive reward early gradually shifted to shorter reaction times with training, whereas the cohort with fixed and later reward timing tended to maintain late lick onsets (Fig. 8*A*,*C*,*D*; average reaction times for the last 100 reward trials = 0.65 ± 0.07 s, 0.78 ± 0.12, and 1.78 ± 0.11 s for two cohorts with conditional reward timing and the cohort with fixed reward timing, respectively; *p* = 1.43 × 10^−6^, one-way ANOVA). Third, when the false alarm patterns were analyzed, we found more erroneous licks in the cohorts with early reward (Fig. 8*E*). Altogether, these traits indicate that the conditional reward timing makes mice generate behavioral outputs more readily, indicating a lowering of the internal threshold for generating licks.

**Figure 9.**
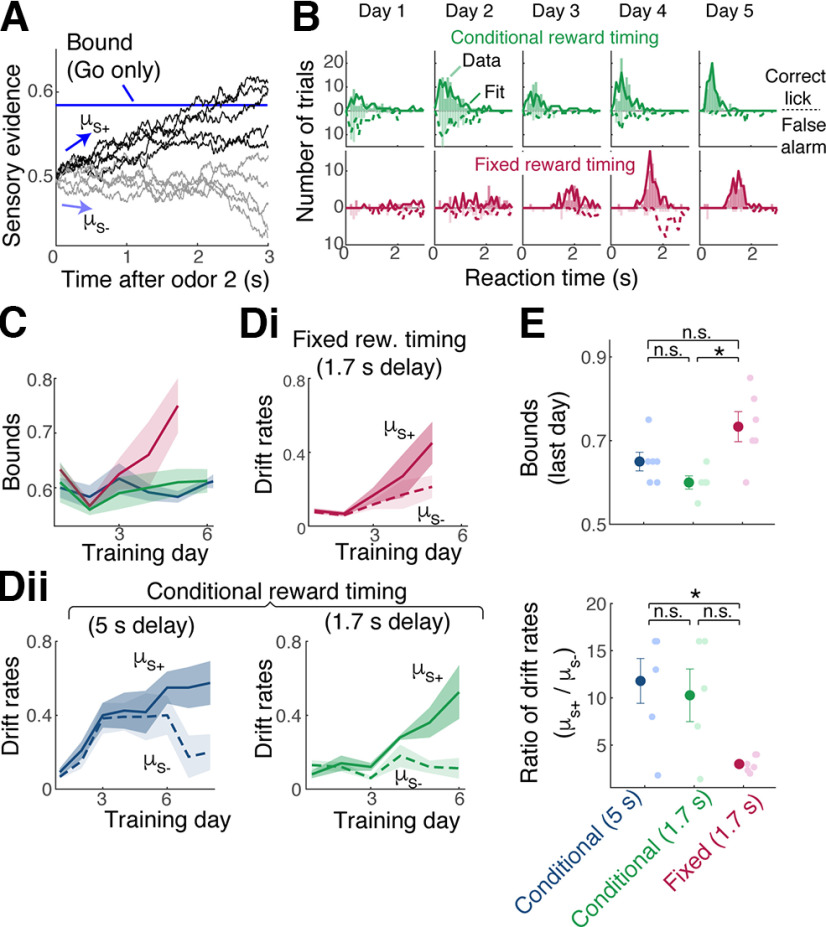
Interplay between reward timing, behavioral threshold, and the discriminability of sensory representations. ***A***, A drift-diffusion model with one bound (blue line) to model the Go/No-Go behavior. Drift rates, µ_s+_ and µ_s−_, are the strengths of momentary evidence following the S+ (nonmatch) stimulus and S− (match) stimulus, respectively. At each time point, a fixed amount of noise is added and accumulated over time (sensory evidence). Reaction time is the time at which the sensory evidence crosses the bound. ***B***, Histograms of reaction times for an example animal over five training days. Observed licks (correct licks are licks on S+ trials; false alarms are licks on S− trials) shown as bars. Simulated result using fitted parameters superimposed with lines (solid lines indicate S+ simulation; dotted lines indicate S− simulation). ***C***, Estimated bounds for cohorts with fixed reward timing (burgundy) and conditional reward timing (green). ***Di***, Estimated drift rates for the cohort with late, fixed reward timing. Rew., Reward. ***Dii***, Estimated drift rates for two cohorts with conditional reward timing. ***E***, Comparison of estimated bounds (top) and the ratio of drift rates (µ_s+_/µ_s−_, bottom) for the three cohorts from the last day of initial training. For decision bounds, *p* = 0.011 and 0.099 across reward timing and *p* = 0.43 for cohorts with same reward timing but different sample-test intervals (one-way ANOVA with *post hoc* Tukey–Kramer tests). For drift rates, *p* = 0.0183 and 0.0639 across reward timing, and *p* = 0.86 for cohorts with same reward timing (one-way ANOVA with *post hoc* Tukey–Kramer tests). Mean and SEM are indicated. n.s. = not significant at the 0.05 level.

To analyze the relationship between the reward timing and internal threshold more rigorously and to explain how a lower behavioral threshold relates to slower learning, we analyzed our data using a simple drift-diffusion model (Ratcliff and McKoon, 2008). This model simulates the internal representation of sensory evidence as an accumulation of noisy diffusion processes (drift rate with noise; Fig. 9*A*). In addition, the time at which this sensory evidence crosses a threshold (bound) models the behavioral reaction times observed. Here, we fit reaction times of the animals and estimated two diffusion rates, corresponding to the strengths of momentary sensory drives for matching and nonmatching odor stimuli (S− and S+, respectively), as well as a single bound (Go bound; Ratcliff et al., 2018). The fitted model captured the tendency for early reaction times, as well as more frequent false alarm occurrences, for the cohort with conditional reward timing and more accurate and later production of anticipatory licks for the cohort with fixed reward timing (Fig. 9*B*).

This analysis revealed how the reward timing may affect the acquisition of a task. First, as indicated by the readiness to generate early anticipatory licks and sporadic licks, earlier arrival of reward tends to lower the decision bound (Fig. 9*C*; mean bounds = 0.65 ± 0.02 and 0.60 ± 0.02 for the two cohorts with early reward vs 0.73 ± 0.04 for the fixed, late reward group; *p* = 0.013, one-way ANOVA; *n* = 5, 6, and 6 mice). Second, we found that the difference in the drift rates associated with the S+ and S− stimuli became greater for the cohort with conditional reward timing as they learned the task (Fig. 9*D*,*E*; ratio of S+ vs S− diffusion rates = 11.8 ± 2.37 and 10.28 ± 2.8 for the two cohorts with conditional reward timing vs 3.00 ± 0.34 for the fixed, later reward group; *p* = 0.017, one-way ANOVA; *n* = 6, 5, and 6 mice). In other words, when the reward arrives early, a consequence is a lowering of the decision threshold, which may require the sensory representations to be more discriminable to achieve the same accuracy. This requirement for more discriminable representation may underlie the slower task acquisition.

**Figure 10.**
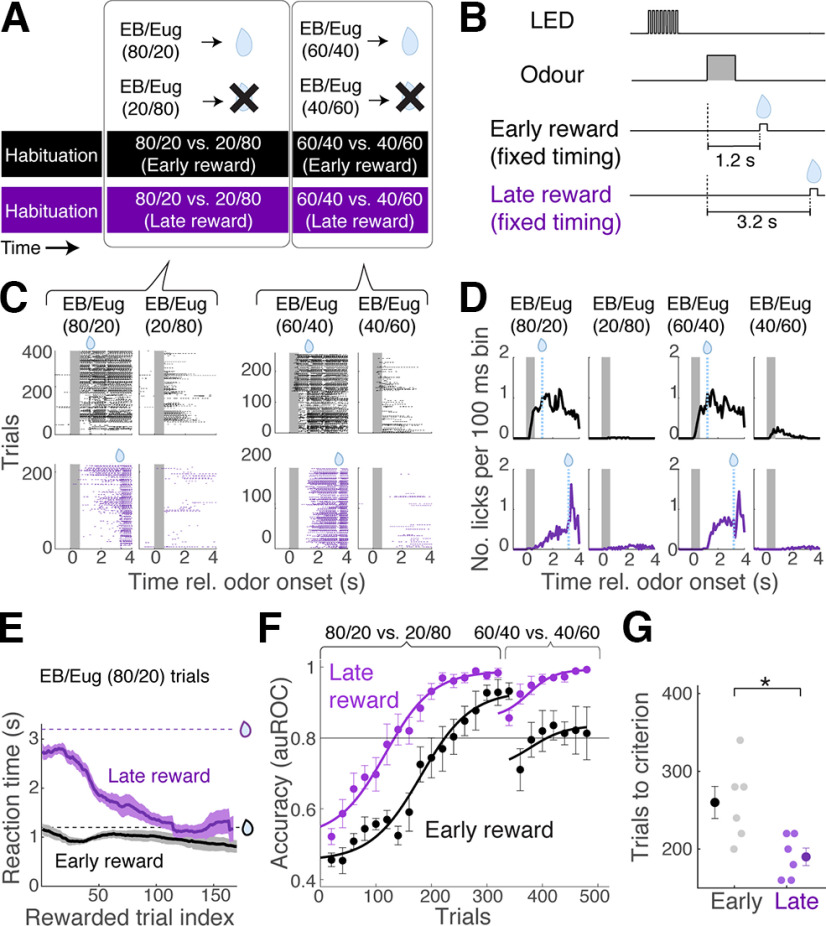
Reward timing effect is likely general; the acquisition of fine olfactory discrimination task is also affected. ***A***, Schematic of the behavioral paradigm. After habituation, head-fixed mice were trained to discriminate between EB and eugenol (Eug) mixtures that differed in the mixed ratios. After reaching the criterion level of performance (80% correct) on 80/20 versus 20/80 discrimination, the mice underwent training for 60/40 versus 40/60 mixture discrimination. ***B***, Trial structure. For the early reward group, the water reward arrived 1.2 s after the onset of odor. For the late reward group, the latency to reward was 3.2 s. ***C***, Lick raster plots from example mice for S+ and S− odors as indicated, for early reward group (top) and late reward group (bottom). ***D***, Peristimulus time histogram of lick occurrences for the last training session for each task. Rel., relative to ***E***, Evolution of lick onset timing for the two cohorts over training. Dotted lines indicate the reward arrival times for the two cohorts; *n* = 6 mice for each cohort. Mean and SEM are indicated. ***F***, Learning curves for the two cohorts. Each data point corresponds to the average accuracy (auROC) for a block of 40 trials sliding every 20 trials. Mean ± SEM is indicated. ***G***, Number of trials taken to reach the criterion level of accuracy (auROC = 0.8) for the initial acquisition; *p* = 0.014, two-sample *t* test; *n* = 6 mice in each cohort. Mean and SEM are indicated.

The above result suggests that a late arrival of a reward allows a more evolved, or discriminable, sensory representation available for task acquisition. We wished to test whether this is a general phenomenon and is relevant for other forms of sensory tasks. To this end, we trained cohorts of head-fixed mice on a Go/No-Go fine olfactory discrimination task. Here, the task was to discriminate between binary olfactory mixtures (Fig. 10*A*,*B*). Both odor mixtures contained ethyl butyrate and eugenol, but the S+ and S− odors differed in the ratios of the component odor concentrations (Koldaeva et al., 2019). One cohort of mice received a reward that arrived 1.2 s after the onset of the S+ odor (early reward group), whereas for the second group, the reward latency was 3.2 s (late reward group) to reproduce the difference in anticipatory lick patterns observed earlier for the conditional reward timing versus late, fixed reward arrival. It should be noted that, here, the timing of the early reward arrival was fixed to rule out a difference in the variability in reward timing contributing to the reward timing effect. After habituation, both cohorts of mice were trained to discriminate the 80/20 (EB/eugenol) mixture versus the 20/80 mixture, followed by discrimination of a more similar pair of stimuli, namely, 60/40 versus 40/60 mixtures (Fig. 10*A*,*B*). The cohort of mice that received the reward with a latency of 3.2 s generated anticipatory licks with a longer latency and more selectively for S+ mixtures than the early reward group from the outset (Fig. 10*C*,*D*). The late reward group reached the criterion level of performance significantly faster than the early reward group. Further, once trained on the 80/20 versus 20/80 discrimination task, the late reward group performed better on the more difficult mixture discrimination. Altogether, these results suggest that the reward timing effect is likely a general phenomenon.

## Discussion

Describing the quantitative relationships between physical stimuli and behavioral change is central to gaining insights into learning. In this study, we demonstrate that simplified training, without punishment, leads to a robust acquisition of the olfactory DNMS task. Further, we identified two aspects of stimulus timing that affect the task acquisition rate. The task acquisition is faster when the sample-test delay interval is shorter and slower when animals could receive a reward earlier.

The effect of the delay interval on the DNMS task acquisition may relate to the degradation or fading of the short-term memory over time, which is a hallmark of working memory (Baddeley, 2012). An underlying neural correlate of short-term information retention is thought to be a stimulus-specific, persistent increase in firing rates in individual neurons. Such activity patterns are prevalent in the prefrontal cortex in animals engaged in delayed response and delayed matching tasks (Fuster and Alexander, 1971; Funahashi et al., 1989; Goldman-Rakic, 1995; Miller et al., 1996; Wu et al., 2020). These activities may be maintained through dynamic interactions within a local network (Goldman-Rakic, 1995; Sreenivasan and D'Esposito, 2019) and possibly across brain regions (Zhang et al., 2019; Wu et al., 2020). However, typically, there is a degradation—or decay in the firing rate—during the delay period, which correlates with task performance (Zhang et al., 2019). Thus, the faded representation may slow down the task acquisition in a manner similar to presenting a weaker stimulus in associative learning (Grant and Schneider, 1948). It should be noted that although this study used a Go/No-Go paradigm, such a weakening of sensory representations is likely to have a similar effect on other instances of delayed (non)match-to-sample, such as the two-alternative forced-choice paradigm.

The detrimental effect of early reward arrival on task acquisition was less expected from the outset. For example, in an influential framework, the temporal contiguity between stimuli and reinforcing signals is described by an eligibility trace, a short-term memory vector that signals a magnitude of reinforcement learning permitted (Klopf, 1972; Barto et al., 1983). As this describes a signal that fades over time, a simple prediction is a slower learning rate with later reward arrival. Further, some studies suggest that waiting longer for more prolonged evidence accumulation can incur a cost to behavioral accuracy (Drugowitsch et al., 2012). However, our finding indicates that there is more to it; when the reward could be delivered earlier, our mice also developed early licking. We interpret this effect on reaction times as lowering the decision bound. A consequence of this lower bound seems that to achieve the same accuracy the momentary strengths of the sensory drive (the drift rates) had to become more divergent. This may be synonymous with improving on a more difficult task. This requirement for more divergent sensory drives required for accurate decision-making may underlie a slower task acquisition. An alternative interpretation of our findings is that a variable reward timing introduces uncertainty. For example, an arrival of a sensory stimulus with an unexpected timing can degrade a behavioral performance (Jaramillo and Zador, 2011). However, it should be noted that in our study mice had control over when the reward should be delivered, resulting in an earlier reaction time. Unexpected stimulus timing, on the other hand, is accompanied by a slower reaction time (Jaramillo and Zador, 2011). Further, using a fine olfactory discrimination task, we found that an early reward timing has a detrimental effect on learning even when the reward timing is fixed. We do not, however, completely rule out the effect of uncertainty, given that the DNMS and fine discrimination tasks likely involve different circuit processing.

Although the drift-diffusion model was instrumental in narrowing potential factors involved, what might the abstract, computational terms correspond to physiologically? Several studies have reported ramping neuronal activities in prefrontal and frontal cortices, for example, in monkeys discriminating and reporting the direction of random dot motions with saccades after delays (Shadlen and Newsome, 1996; Roitman and Shadlen, 2002; Ding and Gold, 2012). Furthermore, the slopes of such ramping activity are known to increase with the coherence of motion or the strength of stimuli (Shadlen and Newsome, 1996; Roitman and Shadlen, 2002; Ding and Gold, 2012). These suggest that the drift rates of the model may be interpreted in terms of mechanisms that lead to action potential generation in relevant prefrontal cortex neurons, for example, the firing rates of neurons presynaptic to these neurons.

In the case of the olfactory delayed matching to sample, a previous study suggested that the anterolateral motor (ALM) cortex may contain neurons that report the match/nonmatch of sample and test odors (Wu et al., 2020). According to this model, the persistent, stimulus-specific activity in response to the sample odor is maintained through recurrent interactions involving many sensory cortices, which is used to compare against the test odor identity to compute the match in ALM. We, therefore, speculate that the drift rate may correspond to this process. Furthermore, with learning, the selectivity of choice-related activity may undergo refinement, just as sensory representations in many brain regions are known to increase in selectivity with the acquisition of a task (Poort et al., 2015).

In summary, our results indicate two ways in which stimulus timing affects stimulus encoding in relation to the acquisition of a DNMS task—retention of sensory information over time and discriminability of match-related signals required for accurate behavioral performance. The efficient DNMS training paradigm we describe here, in turn, may accelerate the investigations of underlying neural mechanisms.
